# Differential Associations of Inflammatory and Endothelial Biomarkers with Disease Activity in Rheumatoid Arthritis of Short Duration

**DOI:** 10.1155/2014/681635

**Published:** 2014-03-03

**Authors:** Ewa Klimek, Anna Skalska, Beata Kwaśny-Krochin, Andrzej Surdacki, Joanna Sulicka, Mariusz Korkosz, Danuta Fedak, Izabella Kierzkowska, Barbara Wizner, Tomasz K. Grodzicki

**Affiliations:** ^1^Department of Internal Medicine and Gerontology, Jagiellonian University Medical College/University Hospital, ul. Śniadeckich 10, 31-531 Cracow, Poland; ^2^Department of Rheumatology and Balneology, Jagiellonian University Medical College/University Hospital, ul. Śniadeckich 10, 31-531 Cracow, Poland; ^3^2nd Department of Cardiology, Jagiellonian University Medical College/University Hospital, ul. Kopernika 17, 31-501 Cracow, Poland; ^4^Division of Rheumatology, Department of Internal Medicine and Gerontology, Jagiellonian University Medical College/University Hospital, ul. Śniadeckich 10, 31-531 Cracow, Poland; ^5^Department of Clinical Biochemistry, Jagiellonian University Medical College/University Hospital, ul. Kopernika 15b, 31-501 Cracow, Poland

## Abstract

*Objectives*. To estimate endothelial dysfunction in patients with rheumatoid arthritis (RA) of short duration in relation to disease activity based on the assessment of 28 joints (DAS28). *Methods*. We studied 29 patients (22 women, mean age 41 (SD, 9) years) with RA of short duration and 29 healthy controls. The RA subjects were divided into those with low (DAS28: 2.6–5.1, *n* = 18) or high (DAS28 > 5.1, *n* = 11) disease activity. Exclusion criteria included clinically overt atherosclerosis and other coexistent diseases. Biochemical markers of inflammatory activation and endothelial dysfunction were measured. *Results*. There were no significant intergroup differences in the majority of classical cardiovascular risk factors. High-sensitivity C-reactive protein, tumor necrosis factor-**α**, and interleukin-6 were increased in RA subjects. Compared to the controls, levels of soluble vascular cell adhesion molecule-1, von Willebrand factor, and pentraxin-3 were significantly elevated in RA subjects with low disease activity, exhibiting no further significant rises in those with high disease activity. Asymmetric dimethyl-L-arginine, soluble E-selectin, monocyte chemotactic protein-1, and osteoprotegerin were increased only in RA patients with high disease activity. *Conclusions*. Our findings might suggest a dissociation of pathways governing generalized and joint-specific inflammatory reactions from those involved in endothelial activation and inflammation within the vascular wall.

## 1. Introduction

Patients with early rheumatoid arthritis (RA) exhibit increased morbidity and mortality due to cardiovascular (CV) diseases [1]. Increased incidence of CV events in RA is mainly a consequence of accelerated atherogenesis [2] that cannot fully be explained by traditional CV risk factors [3, 4]. Accelerated atherogenesis accompanying RA is linked to endothelial activation and dysfunction [5] that has been observed already in patients with early RA [6–8]. Endothelial dysfunction is considered a consequence of a corollary of multiple interactions including classical CV risk factors, genetic predisposition and polymorphisms, chronic inflammation, oxidative stress, and metabolic abnormalities [2, 9, 10]. Among the above mentioned mechanisms, elevated inflammatory activity appears a major contributor to endothelial dysfunction in patients with RA [11–13]. Proinflammatory cytokines such as tumor necrosis factor alfa (TNF-*α*), interleukin 1*β* (IL-1*β*), and interleukin-6 (IL-6), generated within the inflamed synovium of RA patients, are released into the systemic circulation and putatively affect endothelial cells [11]. Endothelial dysfunction/activation is reflected by high levels of leukocyte adhesion molecules (e.g., vascular cell adhesion molecule-1 (VCAM-1), intercellular adhesion molecule-1 (ICAM-1), and E-selectin), increased level of von Willebrand factor (vWf), and a shift towards prothrombotic activity of the endothelium. Endothelial dysfunction is largely mediated by reduced nitric oxide (NO) bioavailability with a probable contribution of increased levels of asymmetric dimethylarginine (ADMA), an endogenous inhibitor of NO synthesis.

To the best of our knowledge, the association of endothelial dysfunction with clinical and biochemical indices of disease severity has not been extensively studied in patients with RA of short duration so far. Therefore, our goal was to estimate the magnitude of endothelial dysfunction in patients with RA of short duration in relation to the degree of systemic inflammatory activation and the well-recognized disease activity score based on the assessment of 28 joints (DAS28).

## 2. Methods

### 2.1. Study Population

We studied 29 adult patients (22 women, 7 men, mean (SD) age 41 (9) years), with RA of short duration recruited from the group of patients with arthritis referred to the Outpatient Rheumatology Clinic of the Department of Internal Diseases of the University Hospital in Krakow. The diagnosis of RA was established according to the revised 1987 American College of Rheumatology (formerly the American Rheumatism Association) criteria [14]. The patients have had disease duration of ≥6 weeks, have not been treated with any biological or non-biological disease-modifying anti-rheumatic drugs (DMARDs), have been without any treatment, or have been receiving a stable dose of non-steroidal anti-inflammatory drugs (NSAIDs) and/or steroids for at least 4 weeks prior to enrollment in our study. Treatment with DMARDs was given to all patients with diagnosis of RA according to current guidelines directly after completion of the study protocol. For all RA patients, DAS28 including high-sensitivity C-reactive protein (hsCRP), the tender joint count (28 joints), the swollen joint count (28 joints), and the patient's assessment of global well-being (100 mm visual analogue scale-VAS) was calculated [15]. We divided patients with RA according to value of DAS28 into two groups: with high and low disease activity with a cut-off point of DAS28 of 5.1, subjects with a DAS28 below 2.6 were excluded from the calculation.

Additionally, we studied 29 healthy control subjects (13 women, 16 men, with the mean age of 32 (SD, 8) years), recruited mainly among personnel of our hospital, their relatives, and friends.

Exclusion criteria—common for both groups—included clinical evidence of atherosclerotic CV disease (i.e., coronary artery disease, history of acute coronary syndrome, stroke/transient ischemic attack, peripheral artery disease, and symptomatic carotid artery stenosis), uncontrolled/untreated hypertension, diabetes, an estimated glomerular filtration rate (eGFR) <60 mL/minute/1.73 m^2^ of body surface area (calculated using the modified Modification of Diet in Renal Disease study [16] equation), chronic or acute inflammatory diseases, a history of neoplastic diseases within 5 years after treatment termination, and current therapy with DMARDs.

### 2.2. Study Protocol

Patients were recruited from July 2009 to June 2011. The procedure was carried out in the morning in the Department of Internal Diseases of the University Hospital in Krakow. The subjects had been previously asked to refrain from eating, smoking, and alcohol or caffeine consumption for at least 12 h. Clinical data including smoking status, family history of premature CV, coexistent diseases, and medication were collected according to a prespecified questionnaire. Blood pressure was measured 3 times on the left arm after 5 minutes of rest in a sitting position; values from 2 last readings were averaged. Mean arterial pressure (MAP) was calculated using the equation: [diastolic blood pressure + 1/3 (systolic blood pressure − diastolic blood pressure)]. Anthropometric measurements including weight, height, and waist circumference were taken and body mass index (BMI) (body mass [kg]/height [m]^2^) was calculated. Then all participants underwent blood sampling for biochemical assays. The study protocol was approved by the Bioethical Committee of Jagiellonian University and written informed consent was obtained from all participants.

### 2.3. Biochemical Assays

Blood samples were taken from the left antecubital vein. Serum lipids (total cholesterol (TC), LDL-cholesterol (LDL-C), HDL-cholesterol (HDL-C), triglycerides (TG)), and glucose and creatinine levels were measured with an Hitachi 917 analyzer (Roche Diagnostics, Hitachi Ltd., Japan) using standardized laboratory techniques. The erythrocyte sedimentation rate (ESR) was determined in whole blood. High-sensitivity C-reactive protein (hsCRP) was measured with immunonephelometry (Nephelometer BM II, Siemens Healthcare Diagnostics Inc., USA). Estimated GFR (eGFR) was assessed by the MDRD equation [16]. Plasma fibrinogen was measured with an BCS analyzer (Siemens Healthcare Diagnostics Inc., USA).

Plasma for additional biochemical analyses was separated and frozen at −70°C until being assayed. By means of enzyme-linked immunosorbent assays (ELISA), levels of cytokines and adhesion molecules were measured: IL-6 (Human IL-6 Immunoassay Quantikine HS), TNF-*α* (Human TNF-*α* Immunoassay Quantikine HS), monocyte chemotactic protein-1 (MCP-1) (Human CCL2/MCP-1 Immunoassay Quantikine), soluble forms of vascular cell adhesion molecule-1 (sVCAM-1) (Human sVCAM-1 Immunoassay Quantikine), soluble forms of E-selectin (sE-selectin) (sE-selectin/CD62E Immunoassay)-(all above: R&D Systems, Abingdon, UK), additionally vWf (vWf, Asserachrom vWF:Ag)-Diagnostica Stago S.A.S., Asnieres-sur-Seine, France. Moreover with the use of ELISA, we measured levels of pentraxin 3 (PTX3) (Human Pentraxin 3/TSG-14 Immunoassay, R&D Systems, Abingdon, UK), osteoprotegerin (OPG) (Human Osteoprotegerin ELISA BioVendor, Brno, Czech Republic), ADMA, symmetric dimethyl-L-arginine (SDMA) (DLD Diagnostika GmbH, Germany), and cystatin C (ALLmed Diagnostics). Homocysteine was measured by high-performance liquid chromatography (HPLC).

In RA patients, rheumatoid factor (RF) was determined by immunoturbidimetric assay (APTEC Diagnostics nv., ALLmed Diagnostics) and anti-cyclic citrullinated peptide antibodies (aCCP) with ELISA (QUANTA Lite CCP 3.1 IgG/IgA ELISA, INOVA Diagnostics, Inc., San Diego, USA).

### 2.4. Statistical Analysis

Data are reported as mean (SD) or median (interquartile range, IQR) unless otherwise indicated. Proportions were compared using chi-square test or Fisher's exact test. The accordance with a normal distribution was tested by Shapiro-Wilk and Kolmogorov-Smirnov tests. For intergroup comparisons between control subjects and RA patients, Student's *t*-test or Mann-Whitney test was used where appropriate. General Linear Models (GLM) procedure was used for age-sex adjusted comparisons between the controls and RA subjects. Homogeneity of variances was verified by Levene's test and in appropriate cases,Welch correction was applied.

GLM were used to test unadjusted and age-sex adjusted differences between the three groups: non-RA and RA subjects with low and high activity of disease-type III sum of square was used. The Bonferroni test was performed for post hoc comparisons. Spearman rank correlations were applied to test associations between systemic inflammatory markers (ESR, hsCRP, TNF-*α*, and IL-6) and biochemical measures of endothelial activation (vWf, MCP-1, ADMA, sVCAM-1, sE-selectin, OPG, and PTX3) in either non-RA and or RA subjects.

Two-tailed *P* values of less than 0.05 were considered statistically significant. Statistical analysis was performed by SAS software v. 9.3 (SAS Institute, Cary, NC, USA).

## 3. Results

*Traditional clinical and biochemical CV risk factors* are presented in Table 1. Patients with RA in comparison to control subjects were older and predominantly female, so the results were adjusted for age and sex. We observed no significant differences between RA patients and the control group in the majority of classical atherosclerosis risk factors (i.e., blood pressure, LDL, TG and glucose levels, BMI, and waist circumference) with the exception of smoking, TC and HDL levels, and positive family history of premature CVD. In addition, patients with RA had significantly higher levels of fibrinogen and cystatin C, lower creatinine, and higher eGFR, but similar homocysteine concentrations as compared to control subjects. Looking for the relationship between lipids and inflammatory markers (ESR, hsCRP, TNF-*α*, and IL-6) and between lipids and biochemical measures of endothelial activation (vWf, MCP-1, ADMA, sVCAM-1, sE-selectin, OPG, and PTX3), we only found positive correlation between level of total cholesterol and vWF (*r* = 0.424, *P* < 0.05), level of LDL and vWF (*r* = 0.453, *P* < 0.05) and negative correlation between level of total cholesterol and IL-6 (*r* = −0.383, *P* < 0.05).

*Biochemical markers of inflammatory activity and endothelial activation* are presented in Table 2. In RA patients, we observed highly significantly elevated levels of inflammatory markers. With regard to markers of endothelial dysfunction/activation, plasma levels of vWf, sVCAM-1, MCP-1, and PTX3 were significantly increased in RA patients, whereas differences in the concentrations of sE-selectin were weakened after the standardization of the results to gender and age. Similarly, as sE-selectin, differences in the value of ADMA, ADMA/SDMA ratio and in concentration of OPG were weakened after the standardization of the results to gender and age. Looking for the relationship between systemic inflammatory markers (ESR, hsCRP, TNF-*α*, and IL-6) and biochemical measures of endothelial activation (vWf, MCP-1, ADMA, sVCAM-1, sE-selectin, OPG, a and PTX3), we found positive correlations between ESR and vWf (*r* = 0.4; *P* < 0.05) and between hsCRP and sE-selectin (*r* = 0.42; *P* < 0.05) in RA patients but not in the control group. Additionally in RA patients OPG correlated positively with sVCAM-1 (*r* = 0.52; *P* < 0.05) and with sE-selectin (*r* = 0.62; *P* < 0.05), in contrast to control group. Taking into consideration associations between DAS28 and mentioned above biochemical measures of endothelial activation, we observed positive correlation only between DAS28 and levels of vWf (*r* = 0.39; *P* < 0.05).

*Comparisons of RA patients divided according to disease activity with the reference to control subjects* are presented in Tables 3 and 4. In RA patients with high disease activity levels of inflammatory markers (ESR, hsCRP, TNF-*α*, IL-6), fibrinogen, sE-selectin, ADMA, and ADMA/SDMA ratio were significantly higher than in those with a low disease activity, whereas concentrations of sVCAM-1, vWf, and PTX3 were significantly elevated in RA subjects with a low disease activity versus control subjects, exhibiting no significant further rises in those with a high disease activity. As compared to the controls, we observed a significant increase in the concentrations of fibrinogen, ADMA, MCP-1, sE-selectin, and OPG exclusively in the RA patients with high disease activity. We observed also lower levels of TC, HDL, and LDL-C in the group with high disease activity in comparison to those with low disease activity and control subjects.

## 4. Discussion

### 4.1. Biochemical Markers of Endothelial Dysfunction 

Patients with RA of short duration exhibited biochemical indices of endothelial dysfunction that is highly significantly increased levels of vWf, MCP-1, and sVCAM-1. This was observed despite no differences in the majority of classical atherosclerotic risk factors (i.e., blood pressure, TG, LDL-C and glucose levels, BMI, and waist circumference) with the exception of smoking, TC and HDL-C level, and positive family history of premature CV disease. Increased prevalence of smoking is of notice in RA group and is undoubtedly a major risk factor for CV in healthy subjects [17] but also takes part in pathogenesis of RA [18]. On the other hand, it has been shown that smoking has a smaller impact on the development of CV diseases in the RA group than in the control group in long-term observations [4]. Patients with active untreated RA usually have reduced TC, LDL-C, and HDL-C levels [19] which is partially consistent with our results. Decreased levels of cholesterol in active and untreated disease may be the result of inflammation, as we observed a negative correlation between total cholesterol and Il-6 in RA patients. Nevertheless lipids may have paradoxical associations with the risk of CV diseases in RA, and lower TC and LDL-C levels are associated with increased CV risk [20]. However, in our study we observed positive correlation between vWF, a marker of endothelial dysfunction, and levels of TC and LDL-C. Additionally, RA patients had elevated cystatin C, whose emerging role as a marker of atherosclerotic arterial disease and CV risk has been pointed out [21]. We have shown, in our previous study, that cystatin C is significantly associated with the probability of pathological thickening of the common carotid artery wall [22].

### 4.2. Relations between Inflammatory and Endothelial Markers

As proinflammatory cytokines and metabolic abnormalities associated with systemic inflammation are considered one of principal mechanisms leading to endothelial dysfunction in patients with RA [2, 11], we looked for the relationship between systemic inflammatory markers (ESR, hsCRP, TNF-*α*, and IL-6) and biochemical measures of endothelial activation (vWf, MCP-1, ADMA, sVCAM-1, sE-selectin, OPG, and PTX3). We found positive correlations between ESR and vWf and between hsCRP and sE-selectin. Additionally in RA, we observed rises in OPG and PTX3-novel predictors of CV disease [23, 24]. The prognostic value of both PTX3 [25] and OPG [26] had been independent of hsCRP, which is consistent with the lack of correlation with hsCRP also in our study. However, OPG potentiates TNF-*α*-induced endothelial expression of VCAM-1, intercellular adhesion molecule-1 (ICAM-1), and E-selectin [27], which is in agreement with our observations, that is positive correlation between OPG and concentrations of sVCAM-1 and sE-selectin in RA patients, probably corresponding to endothelial activation.

### 4.3. Mechanistic Considerations

It is noteworthy that, despite pronounced rises in hsCRP, TNF-*α*, and IL-6 in RA patients with a higher value of DAS28 compared to the remainder, we observed no significant differences in sVCAM-1, MCP-1, vWf, PTX3, and OPG, that is, putative markers of localized inflammation and endothelial activation, between subgroups of RA patients stratified according to DAS28. These findings might suggest dissociation of pathways governing generalized and joint-specific inflammatory reactions from those responsible for inflammatory activation within the vascular wall. Accordingly, we observed that a higher disease activity was not associated with concomitant further rises in vascular biomarkers (sVCAM-1, vWf, and PTX3). No significant correlation between DAS28, CRP and ESR, and serum levels of soluble adhesion molecules, considered as biomarkers of endothelial cell activation, observed before or after an anti-TNF-alpha-monoclonal antibody-infliximab infusion was also found in a series of 34 patients with severe and active disease (mean DAS28 4.27) undergoing treatment with this biologic agent [28].

Reports on the association between inflammatory activation and endothelial dysfunction are inconsistent. Sattar et al. proposed that increased levels of circulating inflammatory mediators might cause activation and damage of endothelial cells in patients with RA [11]. While a positive relationship between markers of inflammation and endothelial dysfunction was reported by some authors [8, 12, 29–31], others showed no associations between endothelial dysfunction and elevated systemic markers of inflammation both in early [6] and long-term RA [32]. Sandoo et al. reported that ESR, CRP, DAS28, and disease durations were unrelated to endothelial function both in microvascular and in macrovascular beds [32]. However, we observed positive correlations between hsCRP and sE-selectin in patients with RA, which may indicate the connection between systemic inflammation and endothelial dysfunction, as was shown in another study [30]. Undoubtedly, non-specific inflammatory markers have multiple potential detrimental effects on endothelial activation and dysfunction. C-reactive protein, a well-established cardiovascular biomarker [33], exerts also an activity by itself. Besides promoting increased expression of adhesion molecules (VCAM-1, ICAM-1, and E-selectin) on endothelial surface, CRP increases monocyte adhesion and migration, stimulates the synthesis of chemotactic factors (MCP-1), and induces endothelial secretion of other proinflammatory factors (NF-*κ*B, IL-6, and IL-8) [34]. Both CRP [35] and TNF-*α* [36] inhibit NO bioavailability. TNF-*α* blocks the activation of endothelial NO synthase (eNOS) by interfering with the phosphorylation of protein kinase Akt [37], diminishes eNOS expression [36], and impedes degradation of ADMA, an endogenous inhibitor of eNOS [38]. An evidence of the harmful effects of TNF-*α* on endothelium may be a study, in which it has been shown that different anti-TNF-alpha drugs have shown to improve endothelial function in RA with severe disease. This improvement was associated with a decrease of inflammation manifested by reduction of DAS28 and CRP [31, 39].

The crucial role in joint inflammation has been postulated for TNF-*α* [40] and IL-6 [41]. There is some evidence that the role played by these cytokines in RA pathogenesis might differ. Dessein and Joffe reported significant reductions of IL-6 after 2 weeks of high-dose intra-articular methylprednisolone combined with DMARDs, associated with a decrease in markers of endothelial dysfunction, without significant changes in other inflammatory cytokines such as IL-1 and TNF-*α* [42]. The observed discrepancies in the studies focused on the relationship between endothelial dysfunction and inflammation may be in part due to single determination of these biomarkers without their repeated assays over time [43].

### 4.4. Vascular Biomarkers in relation to Disease Activity

The lack of significant rises in vascular biomarkers (sVCAM-1, vWf, and PTX3) with increasing disease activity in our patients with RA may suggest that the presence of RA itself-rather than the magnitude of concomitant inflammatory activity-underlies endothelial dysfunction. This concept is in agreement with the results of Vaudo et al., who observed endothelial dysfunction in young-to-middle-aged RA patients with long-term disease duration without traditional CV risk factors, receiving DMARDs, exhibiting a low disease activity prior to study enrollment [44]. To the best of our knowledge, our study is the first to show this phenomenon in RA of short duration. Interestingly, according to our findings, lack of association between flow-mediated endothelium-dependent vasodilatation and biomarkers of endothelial dysfunction was also observed in long-standing RA patients with active disease despite receiving periodical anti-TNF-alpha-infliximab therapy [45]. The lack of significant relation between the majority of inflammatory markers and endothelial dysfunction may result also from complex interactions between different mechanisms involved in endothelial injury, such as—besides classic cardiovascular risk factors and chronic inflammation-genetic predisposition, prooxidative stress, prothrombotic state, and metabolic abnormalities. Nevertheless, the lack of longitudinal assays in our cross-sectional study design limits mechanistic interpretation of our results.

On the other hand, it is noteworthy that, in contrast to other endothelial markers, plasma ADMA and sE-selectin (correlated positively with hsCRP) were elevated only in the RA subjects with higher values of DAS28, that may indicate on their selective association with disease activity. Nonetheless, previous reports on the topic are inconsistent. Unlike Kwaśny-Krochin et al. and Sandoo et al. [46, 47], in the majority of studies, no relationship between ADMA and DAS28 or CRP levels in RA was found [48–52]. Although both Kwaśny-Krochin and Sandoo revealed significant positive correlations between CRP, DAS28, and ADMA, in contrast to our study, they examined the patients with long-lasting RA (respectively mean 8.1 and 7.6 years).

A possible mechanism linking increased ADMA with the presence of inflammation can be a reduced activity of dimethylarginine dimethylaminohydrolase (DDAH) due to elevated TNF-*α*. Accordingly, a decrease in ADMA levels might be expected to accompany a fall in disease activity; nevertheless, reports on early RA patients provided conflicting results. Although Di Franco et al. observed significant decreases in both DAS28 and ADMA level after antirheumatic treatment [52], others have demonstrated no drops in ADMA concentrations in spite of a reduction of disease activity in response to DMARDs, including TNF-*α* antagonists [47, 53]. The inconsistency is suggestive of the notion of a complex net of pathways underlying increased ADMA levels such as decreased expression of DDAH [38] and overexpression of protein arginine type I *N*-methyltransferases in the presence of enhanced oxidative stress and hypoxia in the inflamed synovium [54], as well as possibly increased endothelial cell turnover and the consequent liberation of free ADMA during protein catabolism [5, 55], known to be potentiated in insulin-resistant states [56]. As Dimitroulas et al. have recently described an association between ADMA and insulin resistance in RA [57], elevated ADMA in RA subjects with high disease activity can impair glucose homeostasis with subsequent prognostic effects, analogously to the predictive value of ADMA in future decline of glucose tolerance in stable coronary artery disease [58]. On the other hand, a recent study showed a negative correlation between ADMA levels and total cholesterol and LDL-C in a series of patients with ankylosing spondylitis undergoing period anti-TNF-alpha-monoclonal antibody therapy that at the time of the study had low disease activity [59]. Despite significantly lower levels of total and HDL cholesterol and higher levels of ADMA observed in our RA patients and considering contradictory associations of lipids with the risk of CV diseases in RA, we found no correlation between ADMA and cholesterol in the present study.

### 4.5. Study Limitations

Our study has some limitations. First of all our conclusions are constrained by low number of study participants and a cross-sectional design of the study. It makes impossible to follow changes in the relationship between markers of inflammation and endothelial dysfunction in the course of disease. The recruitment of a large group of newly diagnosed RA subjects without DMARDs has been proven difficult; thus, our results should be confirmed in multicenter prospective study. Additionally, significant intergroup differences in age and sex pose another limitation although we adjusted the results for age and gender. In addition, endothelial function was assessed only by serum biomarkers; further studies including different techniques are warranted.

## 5. Conclusions

In summary, our study shows differential associations of inflammatory and endothelial biomarkers with disease activity in RA of short duration. Higher levels of ADMA and sE-selectin observed in patients with high disease activity, contrary to those with low disease activity, may point out the relationship between the severity of inflammatory response and endothelial dysfunction. However, higher levels of vWf, MCP-1, and sVCAM-1 observed in patients with low disease activity exhibiting no further significant rises with an increase in disease activity may suggest a dissociation of pathways governing generalized and joint-specific inflammatory reactions from those responsible for endothelial activation and chronic inflammation within the vascular wall and that the presence of RA itself, rather than the magnitude of concomitant inflammatory activity, contributes to endothelial dysfunction.

The endothelial dysfunction indicates increased risk of atherosclerosis despite early stage of RA. Together with elevated levels of fibrinogen and cystatin C-recognized as markers of CV risk, we found higher concentrations of PTX3 and OPG-novel biomarkers of endothelial activation and predictors of CV disease. Hence, OPG and PTX3 appear that they may be added to biomarkers of vascular involvement in RA of short duration. Due to limitations of our study, the conclusions require further longitudinal studies to identify endothelial markers relevant for future cardiovascular morbidity in RA.

## Figures and Tables

**Table 1:** Clinical characteristics and traditional cardiovascular (CV) risk factors of RA patients and control subjects.

	RA patients (*n* = 29)		Control group (*n* = 29)		*P* value	*P* adjusted^a^
	Nv	Mean (SD)	Nv	Mean (SD)		
Clinical characteristics						
Age, years	29	40.96 (9.45)	29	31.65 (7.64)	**<0.001**	
Female gender, *n* (%)	29	22 (76%)	29	13 (45%)	**0.030**	
Smoking habit, number (%)	28	12 (43%)	29	5 (17%)	**0.045**	
Positive family history of premature CVD, number (%)	28	15 (54%)	29	6 (21%)	**0.013**	
RF positivity, n (%)	29	25 (86%)	—		**NA**	
aCCP positivity, *n* (%)	28	24 (83%)	—	—	NA	
Disease duration, months	29	12 [4; 18]	—	—	NA	
DAS28	29	4.45 (1.53)	—	—	NA	
Steroids, number (%)	29	11 (38%)	—	—	NA	
NSAIDs, number (%)	29	19 (65%)	—	—	NA	
Antihypertensives, number (%)	29	2 (7%)	29	0	0.236	
Traditional CV risk factors						
Systolic blood pressure, mmHg	29	120.79 (17.95)	28	114.96 (12.78)	0.164	0.872
Diastolic blood pressure, mmHg	29	79.86 (7.72)	28	76.75 (6.76)	0.131	0.825
Mean arterial pressure, mmHg	29	93.5 (10.31)	28	89.48 (7.98)	0.106	0.850
Body mass index, kg/m^2^	28	23.45 (4.04)	29	23.24 (2.63)	0.909	0.427
Waist circumference (cm)	28	78.6 (10.13)	28	80.67 (10.92)	0.435	0.149
Glucose, mmol/L	29	4.63 (0.4)	29	4.73 (0.46)	0.366	0.142
TC, mmol/L	27	5.01 (1.2)	28	5.03 (0.73)	0.937	**0.005**
LDL-C, mmol/L	26	3.07 (0.92)	28	2.85 (0.8)	0.366	0.129
HDL-C, mmol/L	26	1.53 (0.42)	28	1.71 (0.49)	0.160	**0.018**
Triglycerides, mmol/L	26	0.98 (0.47)	28	1.02 (0.54)	0.924	0.450
Creatinine, µmol/L	29	59.27 (10.08)	29	69.76 (9.67)	**<0.001**	**0.008**
eGFR, mL/min per 1.73 m^2^of BSA	29	113.11 (25.19)	29	107.68 (17.23)	0.340	**0.009**
Fibrinogen, g/L	29	4.03 (1.51)	29	2.98 (0.7)	**0.001**	**0.011**
Homocysteine, µmol/L	29	10.28 (4.62)	27	10.56 (4.33)	0.571	0.804
Cystatin C, mg/L	29	0.72 (0.16)	26	0.53 (0.1)	**<0.001**	**<0.001**

Data are shown as unadjusted means (SD) or medians [interquartile range, IRQ] or percentages (%). Nv: valid cases; NA: not applicable; RA: rheumatoid arthritis; RF: rheumatoid factor; aCCP: anti-cyclic citrullinated peptide antibodies; DAS28: disease activity score in 28 joints; NSAIDs: nonsteroidal anti-inflammatory drugs; CVD: cardiovascular disease; TC: total cholesterol; LDL-C: low-density lipoproteins-cholesterol; HDL-C: high-density lipoproteins-cholesterol; eGFR: estimated glomerular filtration rate; BSA: body surface area.

^a^Age-sex adjusted *P* value for the defining groups of patients in ANOVA (GLM models)—type III sum of square (SS) was used.

**Table 2:** Biochemical markers of inflammatory activity and endothelial activation.

	RA patients (*n* = 29)		Control group (*n* = 29)		*P* value	*P* adjusted^a^
	Nv	Mean (SD)	Nv	Mean (SD)		
ADMA, µmol/L	29	0.77 (0.2)	28	0.67 (0.18)	**0.056**	0.154
SDMA, µmol/L	29	0.56 (0.16)	26	0.62 (0.18)	0.125	0.111
ADMA/SDMA ratio	29	1.46 (0.57)	26	1.17 (0.47)	**0.045**	0.100
sVCAM-1, ng/mL	29	744.18 (190.08)	28	613.3 (148.4)	**0.002**	**0.048**
MCP-1, pg/mL	29	395.8 (249.3)	28	262.4 (90.73)	**<0.001**	**0.047**
sE-selectin, ng/mL	29	17.65 (8.67)	28	12.45 (8.02)	**0.007**	0.183
vWf, %	29	109.75 (48.6)	27	73.73 (22.39)	**<0.001**	**0.003**
Osteoprotegerin, pmol/L	29	5.18 (1.36)	28	4.01 (1.06)	**<0.001**	0.108
Pentraxin-3, ng/mL	29	0.74 (0.29)	28	0.45 (0.17)	**<0.001**	**<0.001**
ESR, mm/h	28	37.21 (27.44)	29	6.58 (4.79)	**<0.001**	**<0.001**
hsCRP, mg/L	29	15.26 (24.6)	29	0.84 (0.71)	**<0.001**	**0.006**
TNF-*α*, pg/mL	29	2.55 (1.06)	28	1.78 (1.07)	**<0.001**	**0.003**
Interleukin-6, pg/mL	29	8.48 (8.33)	28	0.86 (0.41)	**<0.001**	**0.001**

Data are shown as unadjusted means (SD).

Abbreviations: Nv: valid cases; RA: rheumatoid arthritis; ADMA: asymmetric dimethyl-L-arginine; SDMA: symmetric dimethyl-L-arginine; sVCAM-1: soluble vascular cell adhesion molecule-1; MCP-1: monocyte chemotactic protein-1; sE-selectin: soluble E-selectin; vWF: von Willebrand factor; ESR: erythrocyte sedimentation rate; hsCRP: high-sensitivity C-reactive protein; TNF-*α*: tumor necrosis factor-*α*.

^a^age-sex adjusted *P*-value for the defining groups of patients in ANOVA (GLM models)—type III Sum of Squares (SS) was used.

^‡^ *P* < 0.05 versus control group, ^¥^ *P* < 0.05 versus RA patients with low disease activity in post-hoc analyses for adjusted ANOVA.

**Table 3:** Traditional cardiovascular risk factors according to DAS28.

	Control group (*n* = 29)		RA patients with low DAS28^*∧*^ (*n* = 15)		RA patients with high DAS28^*∧*^ (*n* = 11)		*P* value^ua^	*P* adjusted^a^
	Nv	Mean (SD)	Nv	Mean (SD)	Nv	Mean (SD)		
Age, years	29	31.65 (7.64)	15	39.77 (9.23)*	11	42.9 (9.92)*	**<0.001**	**<0.001**
Smoking habit, number (%)			15	5 (33%)	11	6 (54%)	0.592	
Steroids, number(%)			15	3 (20%)	11	5 (45%)	0.337	
NSAIDs, number(%)			15	8 (53%)	11	8 (72%)	0.551	
Systolic blood pressure, mmHg	28	114.96 (12.78)	15	115.66 (15.98)	11	129.18 (18.53)^∗#^	**0.028**	0.116
Diastolic blood pressure, mmHg	28	76.75 (6.76)	15	78.44 (7.19)	11	82.18 (8.32)	0.122	0.551
Mean arterial pressure, mmHg	28	89.48 (7.98)	15	90.85 (9.54)	11	97.84 (10.46)*	**0.038**	0.210
Body mass index, kg/m^2^	29	23.24 (2.63)	14	23.72 (3.78)	11	23.03 (4.57)	0.906	0.324
Waist circumference (cm)	28	80.67 (10.92)	14	78.76 (8.56)	11	78.36 (12.62)	0.736	0.204
Glucose, mmol/L	29	4.73 (0.46)	15	4.56 (0.32)	11	4.74 (0.51)	0.373	0.307
TC, mmol/L	28	5.03 (0.73)	14	5.26 (1.21)	10	4.59 (1.09)^‡¥^	0.233	**0.002**
LDL-C, mmol/L	28	2.85 (0.8)	14	3.19 (0.93)	9	2.83 (0.92)^‡¥^	0.402	**0.019**
HDL-C, mmol/L	28	1.71 (0.49)	14	1.61 (0.42)^‡^	9	1.40 (0.41)^‡^	0.206	0.055
Triglycerides, mmol/L	28	1.02 (0.54)	14	1.02 (0.49)	9	0.91 (0.45)	0.845	0.400
Creatinine, µmol/L	29	69.77 (9.67)	15	62.91 (9.74)	11	53.35 (7.83)^∗#‡¥^	**<0.001**	**<0.001**
eGFR, mL/min per 1.73 m^2^ of BSA	29	107.69 (17.23)	15	103.01 (24.77)	11	129.63 (15.79)^∗#‡¥^	**0.002**	**<0.001**
Fibrinogen, g/L	29	2.98 (0.7)	15	3.31 (1.14)	11	5.2 (1.31)^∗#‡¥^	**<0.001**	**<0.001**
Homocysteine, µmol/L	27	10.56 (4.33)	15	10.46 (4.69)	11	10 (4.7)	0.941	0.964
Cystatin C, mg/L	26	0.53 (0.1)	15	0.69 (0.14)^∗‡^	11	0.78 (0.16)^∗‡^	**<0.001**	**<0.001**

Data are shown as unadjusted means (SD). ^*∧*^low DAS28 = (2.6–5.1); high DAS28 = (>5.1).

Nv: valid cases; RA: rheumatoid arthritis; DAS28: disease activity score in 28 joints; TC: total cholesterol; LDL-C: low-density lipoproteins-cholesterol; HDL-C: high-density lipoproteins-cholesterol; ESR: erythrocyte sedimentation rate; hsCRP: high-sensitivity C-reactive protein; TNF-*α*: tumor necrosis factor-*α*; ADMA: asymmetric dimethyl-L-arginine; SDMA: symmetric dimethyl-L-arginine; sVCAM-1: soluble vascular cell adhesion molecule-1; MCP-1: monocyte chemotactic protein-1; sE-selectin: soluble E-selectin; vWf: von Willebrand factor. ^ua^unadjusted *P* value in ANOVA (GLM models). *P < 0.05 versus control group and ^#^ *P* < 0.05 versus RA patients with low disease activity in post hoc analyses for unadjusted ANOVA.

^a^Age-sex adjusted *P* value for the defining groups of patients in ANOVA (GLM models)—type III sum of squares (SS) was used. ^ ‡^ *P* < 0.05 versus control group and ^¥^ *P* < 0.05 versus RA patients with low disease activity in post hoc analyses for adjusted ANOVA.

**Table 4:** Biochemical markers of inflammatory activity and endothelial activation according to DAS28.

	Control group (*n* = 29)		RA patients with low DAS28^*∧*^ (*n* = 15)		RA patients with high DAS28^*∧*^ (*n* = 11)		*P * value^ua^	*P* adjusted^a^
	Nv	Mean (SD)	Nv	Mean (SD)	Nv	Mean (SD)		
ADMA, µmol/L	28	0.67 (0.18)	15	0.69 (0.17)	11	0.88 (0.19)^∗#‡¥^	**0.007**	**0.027**
SDMA, µmol/L	26	0.62 (0.18)	15	0.58 (0.18)	11	0.53 (0.13)	0.404	0.220
ADMA/SDMA ratio	26	1.17 (0.47)	15	1.27 (0.4)*	11	1.77 (0.69)^∗‡¥^	**0.006**	**0.020**
sVCAM-1, ng/mL	28	613.3 (148.4)	15	779.98 (203.67)^∗‡^	11	685.61 (156.74)	**0.008**	**0.033**
MCP-1, pg/mL	28	262.4 (90.73)	15	336.13 (87.84)	11	493.47 (379.53)^∗‡^	**0.015**	**0.030**
sE-selectin, ng/mL	28	12.45 (8.02)	15	14.98 (5.8)	11	22.03 (10.9)^∗‡¥^	**0.006**	**0.047**
vWf, %	27	73.73 (22.39)	15	102.76 (44.78)^∗‡^	11	121.19 (54.51)^∗‡^	**0.008**	**0.019**
Osteoprotegerin, pmol/L	28	4.01 (1.06)	15	4.89 (0.98)	11	5.65 (1.76)^∗‡^	**0.001**	**0.038**
Pentraxin-3, ng/mL	28	0.45 (0.17)	15	0.73 (0.27)^∗‡^	11	0.75 (0.31)^∗‡^	**0.001**	**0.002**
ESR, mm/h	29	6.58 (4.79)	14	21.64 (16.94)^∗‡^	11	61.27 (22.93)^∗#‡¥^	**<0.001**	**<0.001**
hsCRP, mg/L	29	0.84 (0.71)	15	4.48 (6.2)	11	32.9 (32.84)^∗#‡¥^	**0.004**	**<0.001**
TNF-*α*, pg/mL	28	1.78 (1.07)	15	2.06 (0.53)	11	3.33 (1.24)^∗#‡¥^	**<0.001**	**<0.001**
Interleukin-6, pg/mL	28	0.86 (0.41)	15	4.54 (5.11)*	11	14.93 (8.72)^∗#‡¥^	**<0.001**	**<0.001**

Data are shown as unadjusted means (SD).

^*∧*^Low DAS28 = (2.6–5.1); high DAS28 = (>5.1).

Nv: valid cases; RA: rheumatoid arthritis; ADMA: asymmetric dimethyl-L-arginine; SDMA: symmetric dimethyl-L-arginine; sVCAM-1: soluble vascular cell adhesion molecule-1; MCP-1: monocyte chemotactic protein-1; sE-selectin: soluble E-selectin; vWF: von Willebrand factor.

^ua^Unadjusted *P* value in ANOVA (GLM models). *P < 0.05 versus control group and ^#^ *P* < 0.05 versus RA patients with low disease activity in post hoc analyses for unadjusted ANOVA.

^a^Age-sex adjusted *P*value for the defining groups of patients in ANOVA (GLM models)—type III sum of square (SS) was used.

^‡^ *P* < 0.05 versus control group and ^¥^ *P* ≤ 0.05 versus RA patients with low disease activity in post hoc analyses for adjusted ANOVA.
